# Changes in glomerular filtration rate and clinical course after sequential doses of carboplatin in children with embryonal brain tumors undergoing autologous stem cell transplantation

**DOI:** 10.1186/s43046-020-00024-6

**Published:** 2020-02-18

**Authors:** Yasser Elborai, Mohammad Almutereen, Ossama M. Maher, Hanafy Hafez, Michelle A. Lee, Leslie Lehmann

**Affiliations:** 1 Pediatric Stem Cell Transplantation Unit, Dana Farber/Children’s Cancer and Blood Disorders Center, Boston, MA USA; 2 Pediatric Oncology Department, National Cancer Institute (NCI), Cairo University, Cairo, Egypt; 3 Pediatric Immunology and Stem Cell Transplant Unit, Queen Rania Children’s Hospital/King Hussein Medical Center/Royal Medical Services, Amman, Jordan; 4 Department of Pediatric Hematology Oncology, Nicklaus Children’s Hospital, Miami, FL USA; 5 Pediatric Oncology/Pediatric Hematopoietic Stem Cell Transplant Unit, Children’s Cancer Hospital Egypt (CCHE-57357), Cairo, Egypt; 6 Marrow and Blood Cell Transplantation Program, Children’s Hospital at Montefiore, Bronx, NY USA

**Keywords:** Carboplatin, Brain tumor, GFR, Renal toxicity, Autologous stem cell transplant (ASCT)

## Abstract

**Background:**

Treatment for malignant embryonal brain tumors in young children usually employs cycles of standardly dosed cisplatinum followed by high-dose carboplatinum-containing conditioning with single or tandem autologous stem cell rescue (HDC-ASCR). High-dose carboplatin is potentially nephrotoxic, and additive platinum exposure may acutely impact renal function. Aiming to determine if decrease in renal function during conditioning assessed prior to each carboplatin dose was associated with acute increases in creatinine, requirement for dialysis or transplant-related mortality (TRM). This was a retrospective study of consecutive patients with medulloblastoma (*n* = 15) / atypical teratoid/rhabdoid tumor (AT/RT, *n* = 5) receiving HDC-ASCR. Fifteen patients underwent 1 HDC-ASCR (carboplatin × 3 doses/ etoposide/ thiotepa) and 5 patients underwent at least 1 of 3 planned tandem HDC-ASCR (carboplatin × 2 doses/ thiotepa). Renal function was assessed by daily creatinine and nuclear medicine glomerular filtration rate (GFR)/ creatinine clearance before each carboplatin dose.

**Results:**

In this cohort of 20 patients, 3 had doses of carboplatin omitted due to decreases in GFR: 1 did not develop nephrotoxicity, 1 experienced nephrotoxicity without need for dialysis, and 1 required dialysis temporarily but recovered renal function. Two patients did not have GFR changes but developed post-ASCR renal failure requiring dialysis and TRM.

**Conclusion:**

Daily assessment of renal function by GFR, prior each dose of carboplatin during HDC-ASCR, will help in protecting the kidney in heavily treated population of oncology/HSCT patients. Although the study had a small number of patients which is a major limitation of the study, but it points to a serious transplant-related morbidity and mortality. So, larger scale studies are needed to clarify the best approach to carboplatin dosing to insure the optimal balance between efficacy and toxicity.

## Background

Tumors arising from the central nervous system (CNS) are the most common pediatric solid tumors [1]. Malignant embryonal brain tumor of childhood includes medulloblastoma, the most frequent, atypical teratoid/rhabdoid tumor (AT/RT) and the primitive neuroectodermal tumors. Advances in multimodality treatment (surgical intervention, radiation therapy, and chemotherapy) have improved overall survival rates. However, mortality remains high, most often due to recurrent disease [2]. Furthermore, morbidity is significant due to the combined effect of these therapies. The impact of radiation therapy on very young children is anticipated to be so profound neurologically that alternative approaches involving high-dose chemotherapy with autologous stem cell rescue (HDC-ASCR) have been employed for these patients to delay or eliminate exposure to CNS radiation. HDC agents are chosen for their ability to reach higher therapeutic levels in the brain, with the goal of overcoming chemotherapy resistance and ultimately leading to improved survival [3]. As a consequence, there is higher exposure systemically to these drugs. After completion of HDC administration, previously collected autologous peripheral blood stem cells are infused as a rescue from hematologic toxicity [4] but other organs remain at risk. Thus, autologous transplant is designed to allow administration of high doses of tumor-directed chemotherapy with expected and rescuable hematologic consequences but also with anticipated risk of toxicity to other organs.

Most high-dose chemotherapy regimens include carboplatin as it is effective against malignant embryonal brain tumors and able to cross the blood brain barrier (BBB) and blood tumor barrier (BTB). Carboplatin, while significantly less nephrotoxic than cisplatin [5], can lead to a cumulative impairment in renal function. This toxic impact on renal function limits the total dose that can be administered and contributes to transplant-related morbidity and mortality [6].

Pediatric patients undergoing HDC-ASCR for malignant embryonal brain tumors are at significant risk of renal dysfunction. Prior to administration of carboplatin, they have received multiple cycles of standard-dose cisplatin-containing therapy for induction [5]. They have also likely been exposed to multiple other nephrotoxic medications for supportive care including anti-bacterial, anti-fungal, and anti-viral agents. Thus, particular attention must be given to protect the renal function of these vulnerable patients. Carboplatin dosing in autologous transplant is typically based upon evaluation of glomerular filtration rate (GFR), which serves as an index of the number of functioning nephrons [7]. Despite basing carboplatin dosing on a direct measurement of renal function, renal toxicity can still occur during HDC-ASCR. Manifestations range from elevations in serum creatinine to frank renal failure with dialysis dependence to death [8]. One contributing factor may be the administration of carboplatin over multiple days during HDC. It is possible that sequential carboplatin doses result in acute changes in renal function that should prompt reductions in subsequent doses. Thus some centers, including our own, obtain nuclear medicine GFR measurements prior to each dose of carboplatin and adjust subsequent doses if the proximal GFR measurement falls below a certain threshold. We here report our experience employing this approach in children receiving single and tandem transplants. We describe the interventions made when GFR decreased as well as patient outcomes. The data highlights the renal vulnerabilities in these children as well as having broader relevance for all patients receiving high-dose nephrotoxic chemotherapy in the context of HDC-ASCTR.

## Methods

### Study population

This retrospective study includes all pediatric patients with malignant embryonal brain tumors who underwent HDC-ASCR at Dana Farber/Children’s Cancer and Blood Disorders Center between June 2006 and November 2011. Institute review board (IRB) approval was obtained for this retrospective chart review study. Data were stored in a password-protected database.

The medical records of 20 consecutive pediatric patients receiving carboplatin-containing HDC-ASCR to treat primary malignant embryonal brain tumors were reviewed. These 20 patients underwent 28 cycles of HDC-ASCR. All had previously received cisplatin-containing standard chemotherapy (with vincristine, etoposide, cyclophosphamide, plus or minus high-dose methotrexate) prior to HDC-ASCR. Patients had nuclear medicine GFR testing (*n* = 19) or 24-h creatinine clearance (24hrCrCl, *n* = 1) performed prior to each dose of carboplatin.

**Fig. 1:**
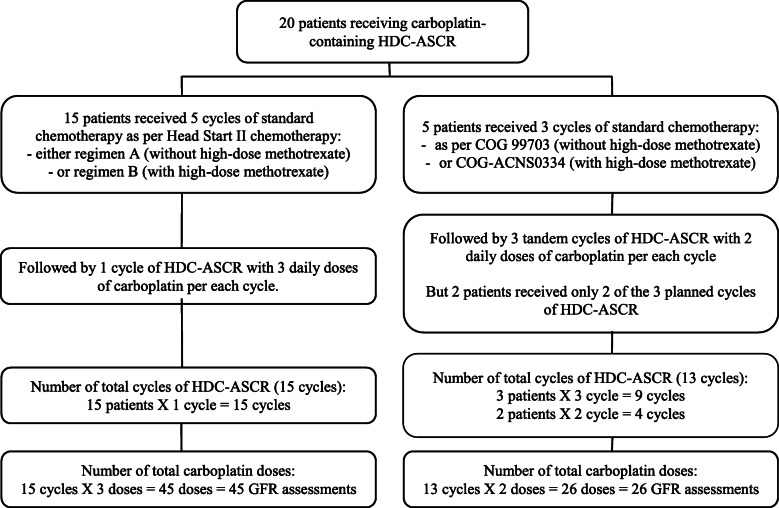
Different approaches using HDC-ASCR to treat pediatric brain tumors

Two different approaches using HDC-ASCR to treat pediatric brain tumors were followed at our institution during this time period (Fig. 1).

Fifteen patients were treated as per Head Start II chemotherapy (either regimen A without [9] or regimen B with high-dose methotrexate [10]). They received 5 cycles of standard chemotherapy followed by 1 cycle of HDC-ASCR with 3 daily doses of carboplatin (dosed as described below) on days − 8, − 7, and − 6; thiotepa 300 mg/m^2^/dose daily on days − 5, − 4, and − 3; and etoposide 250 mg/m^2^/dose daily on days − 5, − 4, and − 3.

Five patients were treated with 3 cycles of standard chemotherapy as per Children Oncology Group—COG 99703 (without high-dose methotrexate) [11] or COG-ACNS0334 (with high-dose methotrexate; not yet published, see ClinicalTrials.gov, Identifier NCT00336024)—planned to be followed by 3 tandem cycles of HDC-ASCR with 2 daily doses of carboplatin (dosed as described below) on days − 4 and − 3 and thiotepa 10 mg/kg/day on days − 4 and − 3. The second and third transplants occurred after neutrophil recovery and at least 21 days from the previous autologous transplant. Three patients received all 3 planned cycles of HDC-ASCR. Two patients received only 2 of the 3 planned cycles of HDC-ASCR.

### Methodology

Per our institutional standard, assessment with nuclear medicine GFR (*N* = 19) or 24hrCrCl (*N* = 1) evaluation occurred within 30 days prior to admission for HDC and was used to calculate the initial carboplatin dose.

When 1 single cycle of HDC-ASCR was undertaken, carboplatin dosing was calculated by 3 separate methods: based on weight (16.7 mg/kg/day), body surface area (500 mg/m^2^/day), and Calvert formula to achieve an area under the curve (AUC) 7 mg/ml/min (only performed in children > 10 kg). The method resulting in the lowest calculated dose was then used. GFR testing prior to each subsequent dose of carboplatin was obtained daily per institutional standard. If the measurement dropped below 70 mg/ml/1.73 m^2^ on any measure, then HDC was delayed until renal function improved. If the delay was greater than 2 days, then no further HDC was administered.

When 3 tandem cycles of HDC-ASCR were planned, carboplatin was given at dose 17 mg/kg/day on days − 4 and − 3 when the initial GFR was greater than 100 ml/min/1.73 m^2^. If the initial GFR was 50–100 ml/min/1.73 m^2^, then the carboplatin dose was to be calculated using the other 2 methods described above and the lowest dose was to be used. If the GFR was lower than 50 ml/min/1.73 m^2^, then carboplatin was to be omitted from the conditioning and thiotepa alone was given.

GFR was determined by plasma clearance of technetium Tc-99m DTPA (diethylene-triamine-pentaacetate), one of the technetium radiopharmaceuticals used in renal imaging. It was assessed by the single injection method, after intravenous administration of 0.456 mCi (Millicurie). Values below (70 ml/min/1.73 m^2^) are considered abnormal [12].

Renal toxicity was assessed by serum creatinine which was monitored daily per institutional standard. Toxicity data were graded according to the criteria of “Common Terminology Criteria for Adverse Events” (CTCAE v. 4.03); grade 1 nephropathy was considered if creatinine is 1.5–2.0 × above baseline, grade 2 nephropathy if creatinine is 2–3 × above baseline, grade 3 nephropathy if creatinine is > 3 × baseline, grade 4 nephropathy if life-threatening consequences or dialysis indicated, and grade 5 nephropathy if death occurred.

## Results

Twenty patients received 28 transplants: 15 patients underwent 1 cycle of HDC-ASCR and 5 patients were planned to receive 3 tandem cycles of HDC-ASCR. Three of these patients received all 3 planned tandem cycles of HDC-ASCR. Two patients received only 2 cycles of HDC-ASCR: one developed grade 4 nephrotoxicity post 2nd cycle of HDC-ASCR requiring hemodialysis but with subsequent recovery, the other patient developed grade 5 nephrotoxicity and died post 2nd cycle of HDC-ASCR (Table 1).

**Table 1:** Patient characteristics

Total number	20
Age	
Median, years (Range)	5 (1–13)
Sex	
Male (%)	9 (45)
Female (%)	11 (55)
Disease at transplant	
Medulloblastoma (%)	15 (75)
AT/RT (%)	5 (25)
Number of transplants	
Total	28
Fifteen patients (single HDC-ASCR for each patient)	15
Three patients (3 out of 3 tandem HDC-ASCR for each patient)	9
Two patients (2 out of 3 tandem HDC-ASCR for each patient)	4

*AT/RT* atypical teratoid/rhabdoid tumor, *HDC-ASCR* high-dose chemotherapy-autologous stem cell rescue

Seventy-one assessments of renal function were performed in this cohort, before admission and within 24 h preceding each subsequent dose of carboplatin. Forty five were done in the 15 patients receiving 1 HDC-ASCR (3 evaluations per transplant) and 26 were done in the patients planned to undergo 3 cycles of HDC-ASCR (2 evaluations for each cycle where 2 patients did not undergo the 3rd cycle). For the 15 patients receiving 1 HDC-ASCR cycle, the median (range) GFR before the first, second, and third doses of carboplatin were 118 (80–284), 144 (75–187), and 111 (75–194) ml/min/1.73 m^2^, respectively. For the five patients scheduled for 3 sequential HDC-ASCR cycles, the median (range) GFR before the first and second doses of carboplatin in the first cycle were 110 (107–140) and 103 (97–140), in the second cycle 120 (91–125) and 103 (62–118), and in the third cycle (3 patients) 116 (103–163) and 110 (97–153) ml/min/1.73 m^2^, respectively (Table 2).

**Table 2:** GFR ranges and changes

1 Cycle HDC-ASCR (*n* = 15), median (range) ml/min/1.73 m^2^	
GFR before 1st dose	118 (80–284)
GFR before 2nd dose	144 (75–187)
GFR before 3rd dose	111 (75–194)
3 Tandem cycles HDC-ASCR, median (range) ml/min/1.73 m^2^	
Cycle1 (*n* = 5)	
GFR before 1st dose	110 (107–140)
GFR before 2nd dose	103 (97–140)
Cycle 2 (*n* = 5)	
GFR before 1st dose	120 (91–125)
GFR before 2nd dose	103 (62–118)
Cycle 3 (*n* = 3)	
GFR before 1st dose	116 (103–163)
GFR before 2nd dose	110 (97–153)

*HDC-ASCR* high-dose chemotherapy-autologous stem cell rescue, *GFR* glomerular filtration rate

### **Outcomes related to GFR and Nephrotoxicity** (Table 3)

#### Patients never having abnormal GFR

Seventeen patients never had a GRF assessment outside of the normal range (< 70 ml/min/1.73 m^2^): fifteen patients still alive and two patients (A & B in Table 3) died due to renal failure.

**Table 3:** GFR as an indicator for renal toxicity

Number of patients	Number of cycles planned	Number of cycles completed	Number of GFR evaluations	Decrement in GFR	Dose modification of carboplatin	Nephrotoxicity	Dialysis	Transplant-related mortality
15	21	21	54	No	No	Not more than grade 1	No	No
1 (A)	Single - 1	1	Baseline 24hrCrCl, then 2 GFR	No	No	Grade 5	Yes	Yes
1 (B)	Tandem - 3	2	4	No	No	Grade 5	Yes	Yes
1 (C)	Single – 1	1	3	Yes	Dose 3 omitted	Grade 1	No	No
1 (D)	Single – 1	1	3	Yes	Dose 3 omitted	Grade 2	No	No
1 (E)	Tandem - 3	2	4	Yes	Cycle 2, dose 2 decreased 50%; Cycle 3 omitted	Grade 4	Yes	No

*24hrCrCl* 24-h creatinine clearance, *GFR* glomerular filtration rate

Fifteen patients (54 evaluations) had GFR values in the normal range at every evaluation and had a benign clinical course. Specifically, no patient in this group had greater than grade 1 nephropathy, needed renal dialysis or experienced transplant-related mortality (TRM). Twelve underwent 1 single HDC-ASCR (36 evaluations), and 3 underwent all 3 planned tandem transplants (18 evaluations). Based on a worsening hearing evaluation, one of these 3 received 50% reduction of the carboplatin dose in HDC-ASCR cycles 2 and 3.

Two patients (A & B in Table 3) ultimately developed severe nephrotoxicity and received renal dialysis preceding death from transplant-related complications.

Patient A underwent 1 single HDC-ASCR cycle. 24hrCrCl was 87 ml/min/1.73 m^2^ before the 1st dose, GFR was 90 ml/min/1.73 m^2^ before the 2nd, and 120 ml/min/1.73 m^2^ before the 3rd. The patient became anuric on day + 1 and experienced grade 5 nephrotoxicity (maximum creatinine 3.6 mg/dl on day + 3, with baseline 0.3). The patient had intracranial infarction, required hemodialysis, and died of multi-organ failure in the context of graft failure and severe infections on day + 38.

Patient B received only 2 out of the 3 planned tandem cycles of HDC-ASCR. GFR evaluations were normal prior to each carboplatin dose for cycle 1, 140 and 140 ml/min/1.73 m^2^. In cycle 2, GFR values also were normal prior to each carboplatin dose, 125 and 118 ml/min/1.73 m^2^. The patient developed grade 5 nephrotoxicity (maximum creatinine 1.8 mg/dl on day + 14, with baseline creatinine 0.2 mg/dl). This occurred in the context of ileus and culture-negative sepsis. The patient required renal dialysis and died from multi-organ failure with intracranial hemorrhage on day + 16.

#### Patients with GFR decrease

Three patients (C, D, & E in Table 3) demonstrated decrements in GFR values resulting in changes in carboplatin dosing; all had at least one dose of carboplatin omitted.

Patient C underwent 1 single cycle of HDC-ASCR. GFR before the 1st carboplatin dose was 98 ml/min/1.73 m^2^, before the 2nd was 121 ml/min/1.73 m^2^, before the 3rd was 84 ml/min/1.73 m^2^. The third dose of carboplatin was omitted because of the decline in GFR from 121 to 84 ml/min/1.73 m^2^, GFR lost around 30% of its previous value, even though GFR not below the previously set threshold of 70 ml/min/1.73 m^2^. Two days later from the omitted dose of carboplatin, the patient developed grade 1 nephrotoxicity (maximum creatinine 0.6 mg/dl on day − 4, with baseline creatinine 0.3 mg/dl) which means that the rapid drop of GFR after 2nd dose of carboplatin was a good and early indicator of nephropathy even before rising of creatinine. The patient did not require dialysis or experience transplant-related mortality.

Patient D underwent 1 single cycle of HDC-ASCR. GFR before the 1st carboplatin dose was 80 ml/min/1.73 m^2^ and before the 2nd was 75 ml/min/1.73 m^2^. The third dose of carboplatin was omitted after discussion even though GFR was not below the previously set threshold of 70 ml/min/1.73 m^2^. But both readings were very close to the threshold with descending trend. Five days later from the omitted dose of carboplatin, the patient developed grade 2 nephrotoxicity (maximum creatinine 1.2 mg/dl on day − 2, with baseline 0.5) which means that the drop of GFR, near the previously set threshold of 70 ml/min/1.73 m^2^, was an early effective indicator of nephropathy even before rising of creatinine. The patient did not require renal dialysis and did not experience transplant-related mortality; creatinine recovered over the following weeks.

Patient E received 2 of 3 planned tandem cycles of HDC-ASCR. GFR evaluations for tandem cycle 1 were normal at 110 and 103 ml/min/1.73 m^2^. During tandem cycle 1, the patient developed HSV lesions treated with acyclovir. After tandem cycle 1, the patient developed cytomegalovirus (CMV) viremia treated with ganciclovir and cytogam. The patient then proceeded to tandem cycle 2. The GFR evaluation before the 1st carboplatin dose was 91 ml/min/1.73 m^2^ and full dose carboplatin was given. The GFR before the second dose was 62 ml/min/1.73 m^2^ which is below the previously set threshold of 70 ml/min/1.73 m^2^, so the carboplatin dose was reduced by 50%. Four weeks later from the reduced dose of carboplatin, the patient developed grade 4 nephrotoxicity (maximum creatinine of 3.6 mg/dl on day + 24, with baseline creatinine 0.6) and required dialysis. During this cycle, the patient also had respiratory failure requiring intubation presumed due to CMV pneumonitis with CMV polymerase chain reaction (PCR) positivity on fluid from brochoalveolar lavage and thus continued ganciclovir therapy. In addition, the patient had Clostridium difficile colitis, urinary tract infection with bacteria and fungi, and signs of thrombotic microangiopathy. Tandem cycle 3 was omitted. The patient did not experience transplant-related mortality and recovered renal function. She died of relapsed disease 4 years after her second cycle of HDC-ASCR.

In the whole cohort, at least one carboplatin dose was omitted in 15% (3 of 20) of patients. One patient experienced grade 1 nephrotoxicity, one experienced grade 2 nephrotoxicity, and one experienced grade 4 nephrotoxicity requiring dialysis but eventually recovering renal function. In two other patients who died with renal failure, the GFR did not predict the subsequent irreversible deterioration of renal function (Table 3). Overall 10% (2 of 20) of patients in this cohort died of transplant-related causes complicated by acute renal failure. One patient was lost to follow-up, eight patients died of disease, and nine out of nineteen patients (47%) are alive with median follow up 8 years (range 5 years to greater than 9 years).

## Discussion

Platinum-based agents are an essential part of therapy in children with malignant embryonal brain tumors including medulloblastoma and ATRT. Children receive cisplatin multiple times during the course of standard dose chemotherapy. Then young children have additional exposure to carboplatin as a component of high-dose chemotherapy followed by autologous stem cell rescue, utilized in an attempt to avoid high-dose radiation therapy in this vulnerable population.

While cisplatin is known to have long-term potentially irreversible renal toxicity, carboplatin was developed to be more protective of the kidney [13]. Yet carboplatin also has been shown to have deleterious effects on kidney function, though those effects are usually reversible [14]. In one study, exposure to carboplatin caused mild GFR impairment (GFR 60–89 ml/min/1.73 m^2^) in only 3% of patients [15]. Bergeron et al. support this conclusion, finding that only one patient out of 30 experienced a decrease in GFR to less than 89 ml/min/1.73 m^2^ as measured by the Schwartz formula [13]. However, these patients did not receive repetitive doses of platinum containing agent cumulating in myeloablative doses of carboplatin. Our results demonstrate that in this setting, carboplatin is associated with renal impairment during HDC as reflected by a decrement in GFR occurring in 15% of our cohort.

The starting carboplatin dose was determined by the initial pre-transplant GFR/24HrCrCl value per institutional protocol. Interestingly, while no subsequent GFR measurement met criteria for omission or dose reduction based on a cutoff of 70 ml/min/1.73 m^2^ or 50 ml/min/1.73 m^2^, 3 patients had carboplatin doses omitted based on clinical judgement that included GFR decrements as well as rising concurrent patient illness and need for other nephrotoxic medications.

There is scant literature on renal toxicity in children undergoing HDC-ASCR for malignant embryonal brain tumors, even though it is appreciated that this group is at high risk of such toxicity given the repetitive platinum exposure. Several studies mention toxic deaths occurring in 5–10% of this population but details are not given [16, 17]. Cheuk et al. reported that 7/13 (54%) of patients developed significant nephrotoxicity with one patient expiring in the early post-transplant period from grade 5 nephrotoxicity [18]. Transplant-related mortality (TRM) in our cohort was 10%, lower than in Cheuk’s study. GFR-based changes may have averted nephrotoxicity and need for dialysis and even TRM, as all three patients for whom GFR decrement prompted carboplatin omission recovered and survived.

While sequential GFR evaluation can help anticipate renal failure and prompt changes in carboplatin dosing that may avert it, carboplatin is not the only contributor to nephrotoxicity during HDC-ASCR. Potential renal insults are multifactorial, including the accumulated toxicity of anti-bacterial, anti-viral, and anti-fungal agents. Such multifactorial renal insults were seen for patient E who experienced bacterial sepsis while being treated with a nephrotoxic anti-viral medication for CMV pneumonitis. Of obvious concern are 2 patients who died of renal toxicity and associated multi-organ failure without appreciable changes in renal function as measured by GFR during the period of carboplatin administration. Both experienced early toxicity and an etiology for the renal failure component was not established.

## Conclusion

It is clear that this heavily treated population of oncology/HSCT patients are at risk of renal compromise. Meticulous renal protection may impact not only acute nephrotoxicity but also transplant-related morbidity and mortality in this population during and after HDC-ASCR. This study has broader relevance to all transplant patients receiving multiple kidney toxic medications as renal complications remain a significant contributor to poor patient outcomes in the transplant setting. The small sample size in this study is a major limitation and precludes a full understanding of the role of frequent GFR measurements in the dosing of medications known to be acutely nephrotoxic. Larger prospective studies will be required to more clearly delineate the best approach to carboplatin dosing and renal protection to achieve the optimal balance between efficacy and toxicity.

## Data Availability

The data that support the findings of this study are available from a password-protected database in Dana Farber/Children’s Cancer and Blood Disorders Center. But restrictions apply to the availability of these data, which were used under license for the current study, and so are not publicly available. Data are however available from the authors upon reasonable request and with permission of Dana Farber/Children’s Cancer and Blood Disorders Center.

## Abbreviations

24hrCrCl: 24-Hour creatinine clearance
AT/RT: Atypical teratoid/rhabdoid tumor
AUC: Area under the curve
BBB: Blood brain barrier
BTB: Blood tumor barrier
CMV: Cytomegalovirus
CNS: Central nervous system
COG: Children oncology group
CTCAE: Common terminology criteria for adverse events
DTPA: Diethylene-triamine-pentaacetate
GFR: Glomerular filtration rate
HDC-ASCR: High-dose chemotherapy-autologous stem cell rescue
IRB: Institute review board
mCi: Millicurie
PCR: Polymerase chain reaction
Tc-99m: Technetium Tc-99m
TRM: Transplant-related mortality
